# Multiple imputation validation study: addressing unmeasured survey data in a longitudinal design

**DOI:** 10.1186/s12874-020-01158-w

**Published:** 2021-01-06

**Authors:** Claire A. Kolaja, Ben Porter, Teresa M. Powell, Rudolph P. Rull, for the Millennium Cohort Study Team, Richard Armenta, Satbir Boparai, Felicia Carey, Toni Rose Geronimo, Isabel Jacobson, Cynthia LeardMann, Rayna Matsuno, Deanne Millard, Chiping Nieh, Anna Rivera, Beverly Sheppard, Daniel Trone, Jennifer Walstrom, Steven Warner

**Affiliations:** 1 Leidos, Inc, 140 Sylvester Road, San Diego, CA 92106 USA; 2 Deployment Health Research Department, Naval Health Research Center, 140 Sylvester Road, San Diego, CA 92106 USA

**Keywords:** Multiple imputation, Cohort study, Survey data, Longitudinal data, Major depressive disorder, Suicidal ideation, Patient health questionnaire

## Abstract

**Background:**

Questionnaires used in longitudinal studies may have questions added or removed over time for numerous reasons. Data missing completely at a follow-up survey is a unique issue for longitudinal studies. While such excluded questions lack information at one follow-up survey, they are collected at other follow-up surveys, and covariances observed at other follow-up surveys may allow for the recovery of the missing data. This study utilized data from a large longitudinal cohort study to assess the efficiency and feasibility of using multiple imputation (MI) to recover this type of information.

**Methods:**

Millennium Cohort Study participants completed the 9-item Patient Health Questionnaire (PHQ) depression module at 2 time points (2004, 2007). The suicidal ideation item in the module was set to missing for the 2007 assessment. Several single-level MI models using different sets of predictors and forms of suicidal ideation were used to compare self-reported values and imputed values for this item in 2007. Additionally, associations with sleep duration and smoking status, which are related constructs, were compared between self-reported and imputed values of suicidal ideation.

**Results:**

Among 63,028 participants eligible for imputation analysis, 4.05% reported suicidal ideation on the 2007 survey. The imputation models successfully identified suicidal ideation, with a sensitivity ranging between 34 and 66% and a positive predictive value between 36 and 42%. Specificity remained above 96% and negative predictive value above 97% for all imputed models. Similar associations were found for all imputation models on related constructs, though the dichotomous suicidal ideation imputed from the model using only PHQ depression items yielded estimates that were closest with the self-reported associations for all adjusted analyses.

**Conclusions:**

Although sensitivity and positive predictive value were relatively low, applying MI techniques allowed for inclusion of an otherwise missing variable. Additionally, correlations with related constructs were estimated near self-reported values. Therefore, the other 8 depression items can be used to estimate suicidal ideation that was completely missing from a survey using MI. However, these imputed values should not be used to estimate population prevalence.

**Supplementary Information:**

The online version contains supplementary material available at 10.1186/s12874-020-01158-w.

## Background

Missing data is a pervasive source of error and bias in prospective cohort studies. Incomplete data from individuals may jeopardize their inclusion in a statistical analysis and thus introduce error, as well as increase uncertainty due to reduced statistical power [1, 2]. In longitudinal cohort studies that involve repeated surveys, questions may be added or removed from a given time point for a variety of reasons, including the reduction of participant burden, application of updated diagnostic criteria for specific conditions, or the assessment of emerging health concerns [3]. The removal of questions from a longitudinal survey introduces a loss of data that restricts researchers’ ability to incorporate this information into analyses. In contrast to public health surveillance and cross-sectional surveys, longitudinal studies have the utility of identifying temporal associations between putative risk factors and outcomes.

One example of a missing data problem arising from a systematic revision of a survey is the depression module of the Patient Health Questionnaire (PHQ-9). This measure is a validated 9-item screening tool for depression in which the ninth item asks about recent thoughts of being better off dead or hurting one’s self [4]. A shortened measure, the PHQ-8, excludes this item. Some large telephone surveys and longitudinal studies have chosen to use the PHQ-8 because of the inherent inabilities to screen participants in real time for eminent self-harm behavior and implement risk management protocols across a large study population [5, 6]. For example, the annual Behavioral Risk Factor Surveillance System, a national telephone survey conducted by the Centers for Disease Control and Prevention, contains the PHQ-8 in the 2006, 2008, and 2010 surveys. In contrast, the National Health and Nutrition Examination Survey (NHANES) has included the PHQ-9 since 2006 [7]. NHANES is a national survey conducted via in-person interviews, which allows for assessment of the last item of the PHQ-9. The Millennium Cohort Study of over 200,000 enrolled service members and veterans, included the PHQ-9 on the first 3 surveys before switching to the PHQ-8 in 2011 due to institutional review board (IRB) requirements to perform risk assessments on all participants who endorse suicidal ideation [8]. This coincided with ethical scrutiny applied to Department of Defense and Department of Veterans Affairs (VA) studies regarding the use of the ninth item of the PHQ-9. VA studies later determined that endorsement of suicidal ideation on the PHQ-9 was a strong predictor of suicide attempt and death over the year following endorsement [9]. This motivated a subsequent VA study to develop a response protocol designed to address reported suicidal ideation among a cohort of almost 15,000 veterans [10].

Multiple imputation (MI) is a statistical approach that employs the observed covariance matrix for variables of interest in order to recover missing information by estimating a plausible set of values. Analyses conducted within each of these imputed data sets can be combined to yield unbiased estimates of true values [11–15]. The fully conditional specification (FCS) MI approach uses a series of univariate models for the conditional distribution of a variable given all other variables listed in the model. Variables are imputed in the order that they are listed, after which the imputed values are saved to the imputed data set [16]. The final imputed data set has a specified number of rows for each observation with complete information. To our knowledge, no previous study has validated this MI technique to impute a factor that is completely missing at one survey wave with the observed covariance structure at another wave in a longitudinal cohort study. Similar to this situation is a study that tested different MI methods to impute factors that are systematically missing for individual participant data meta-analyses [17]. Individual participant data meta-analyses use data from many clinical studies to predict an outcome for multiple factors and summarize the observed associations of those factors. MI can be applied to impute factors for relevant studies that had not assessed all factors of interest. That study compared several types of MI (i.e., traditional, stratified, and multilevel) to complete case analysis. Based on an empirical example and simulation study, they recommended multilevel MI (MLMI) as it preformed the best, even when compared to complete case analysis.

To our knowledge, there is currently no prescribed method for addressing variables that are completely missing at a given longitudinal assessment but had been present for others. Given that related variables will presumably be included on other assessments, associations between the missing variable and observed variables can be determined from surveys in which both are assessed. These associations may be able to impute values on surveys with variables that are completely missing. The current study tested the feasibility of such a technique by imputing suicidal ideation in a large cohort when a single item is completely missing from 1 of 2 longitudinal assessments.

## Methods

### Study population

Data from the Millennium Cohort Study, the largest and longest running prospective cohort study of US service members, was used in these analyses. Participants, selected from active duty and Reserve/National Guard administrative rosters between 2000 and 2010, are followed during their military career through their return to civilian life [18]. At the time of enrollment, participants provided voluntary, informed consent prior to completing the baseline survey and were asked to complete a survey approximately every 3–5 years. These surveys include a wide range of health outcomes and behaviors assessed by standardized instruments such as the PHQ. The study was approved by the Naval Health Research Center IRB (protocol number NHRC.2000.0007). The current analyses included participants who were enrolled in 2001 or 2004 and completed surveys during the 2004–2006 (2004) and 2007–2008 (2007) assessments (*n* = 63,589). There were 560 participants excluded because of missing suicidal ideation responses on the 2007 survey and 1 additional participant was excluded due to missing all PHQ-8 responses on the 2007 survey, yielding a final sample of 63,028 participants.

### Measures

#### Patient health questionnaire

The PHQ-9 is composed of 9 questions that correspond to the Diagnostic and Statistical Manual of Mental Disorders, Fourth Edition (DSM-IV) depression criteria with responses on a 4-point Likert scale from “not at all” to “nearly every day” [6]. Suicidal ideation was ascertained from the ninth item on the PHQ-9, which asks about the frequency in the previous 2 weeks of “thoughts that you would be better off dead or hurting yourself in some way.” Suicidal ideation was assessed in two ways. First, all four levels of the 9th item were assessed. Second, ideation was dichotomized and considered present if the 9th item was reported on “several days” or more. To mimic the scenario of a single item missing entirely on a follow-up survey, self-reported suicidal ideation was set to missing on the 2007 survey.

#### Auxiliary variables for imputation models/covariates

Age, sex, and race/ethnicity variables were obtained from administrative records maintained by the Defense Manpower Data Center (DMDC). Marital status and education were self-reported on the Millennium Cohort survey and backfilled with DMDC data if missing. All other variables (listed below) were assessed using self-reported survey data.

Posttraumatic stress disorder (PTSD) was measured with the PTSD Checklist−Civilian Version (PCL-C), a validated 17-item instrument that measures the severity of PTSD symptoms [19] and has been shown to have good internal consistency in this cohort [20]. Sensitive criteria for PTSD, based on the DSM-IV, were indicated when moderate or greater was endorsed on a minimum of 1 intrusion symptom, 3 avoidance symptoms, and 2 hyperarousal symptoms [21]. Physical functioning was measured using the corresponding 10-item module from the Veterans RAND 36-Item Health Survey [22, 23] that asks about limited ability to perform different activities on a typical day (i.e., vigorous activities like running, lifting groceries, walking more than a mile). Alcohol misuse was assessed from 5 binary alcohol-related problem items on the PHQ (e.g., drank alcohol, when a doctor told you not to, missed or were late to school or work because you were drinking or hung over) [24–26]. Smoking status was categorized into 3 groups (never, former, and current smoker) based on 2 questions of ever smoking at least 100 cigarettes and successful smoking cessation. Never smokers indicated they had not smoked at least 100 cigarettes, former smokers reported smoking at least 100 cigarettes and had successfully quit smoking, and current smokers reported smoking at least 100 cigarettes and had not successfully quit. Average sleep duration was assessed with a single question, “Over the past month, how many hours of sleep did you get in an average 24-hour period?” and categorized into 5 or less hours, 6, 7–9, and 10 or more, based on National Sleep Foundation recommendations [27].

### Statistical analyses

Analyses examined survey data collected in 2004 and 2007. Suicidal ideation on the 2007 survey was set to missing and 100 complete data sets were created using 4 different imputation models. Because the values of suicidal ideation were set to missing for the entire 2007 assessment, MI was not possible using the data in a wide format (i.e., 1 row of data per person). Therefore, surveys were appended so that each participant had one row for the 2004 survey and one row for the 2007 survey (i.e., a long format with 1 row of data per person per time point). The relationships between items at the 2004 survey could thus be used to inform the completely missing item on the 2007 survey.

Naïve values (RAN) were created by randomly assigning suicidal ideation based on the prevalence observed in the sample in 2007 (4.05%). This random generation of suicidal ideation represented values created without any predictive information and thus represented an uninformed baseline that could be improved with MI.

Single level MI (SLMI) was employed to estimate suicidal ideation for all models among all eligible participants. The first model treated suicidal ideation as a dichotomous variable (PHQ-BIN) and included the remaining 8 PHQ items in the imputation model. Because dichotomous suicidal ideation is used in the scoring algorithm and as a predictor/covariate, this model estimated suicidality as it would be used in many analyses. The second model treated suicidal ideation as a 4-level variable (PHQ-ORD) and included the remaining 8 PHQ items in the imputation model. The 4-level imputed ideation variable was then dichotomized in analyses. The third model (ALL-BIN) treated suicidal ideation as a dichotomous variable and included the 8 PHQ items and previously identified factors from the literature: sex, age, race/ethnicity, marital status, education attainment, 10 individual items from the RAND physical functioning module, 17 individual items and PTSD screener from the PCL-C, smoking status, sleep duration, and 5 alcohol use items from the PHQ [28–31]. The fourth model was identical to ALL-BIN, except suicidal ideation was treated as a 4-level variable (ALL-ORD). The PROC MI procedure in SAS (SAS Institute Inc., Cary, NC) was utilized to run MIs using FCS that permits the inclusion of binary and categorical variables. For all models, the other 8 PHQ items were included as 4-level categorical variables. Variables were estimated in the model depending on the format of the variable (e.g., dichotomous, categorical, continuous). Binary and categorical variables were determined using the discriminant function of PROC MI. Sleep duration was included in the imputation models as a continuous variable then categorical for analyses. For PTSD, both the composite variable and the individual items were included in the model. This method allowed for individual items to inform the model (e.g., whether the item “feeling as if your future will somehow be cut short” is more related to suicidality than other PCL-C items).

Initial analyses described the counts and proportions of true negatives, true positives, false negatives, and false positives for imputed and self-reported values of suicidal ideation on the 2007 survey, as well as the prevalence, sensitivity, specificity, positive predictive value (PPV), and negative predictive value (NPV) for each imputation model. All values were calculated separately for each imputed dataset and then pooled across the 100 imputed datasets. Additional analyses calculated the associations between self-reported and imputed values of suicidal ideation and related constructs to ensure that the imputed values maintained their associations with constructs. Separate multinomial logistic regression models estimated the associations of suicidal ideation and the outcomes of sleep duration and smoking status with adjustment for sex, age, race/ethnicity, marital status, and education. The randomly assigned values (i.e., RAN) were not compared with outcomes because they would have been unrelated due to random assignment.

Lastly, we were interested in comparing the difference between the associations (self-reported and imputed values) of suicidal ideation with related constructs. We reported (yes/no) whether the 95% confidence intervals for the associations between the imputed suicidal ideation with the outcomes of interest were overlapping at all with the 95% confidence intervals from the self-reported associations.

Post-hoc multilevel MI (MLMI) analyses were conducted to examine whether the results could be improved from the SLMI models. MLMI allows for associations to be imputed within subjects. Due to computational limitations, these analyses were run among a random subset of 10,000 participants who were eligible for the main analyses. MLMI was implemented by using the JOMOIMPUTE function in the MITML package in R Studio [32]. All factors (i.e., sex, age, race/ethnicity, marital status, education attainment, continuous physical functioning score, PTSD screener from the PCL-C, smoking status, sleep duration, 5 alcohol use items from the PHQ, and the other 8 items from the PHQ depression module) were included in this model as continuous except for dichotomous suicidal ideation. An identical imputation model was run with a SLMI among the same subset of participants to compare the MLMI and SLMI results.

## Results

As previously mentioned, 2553 (4.05%) of 63,028 participants eligible for the main analysis endorsed suicidal ideation as measured by the ninth item on the PHQ-9 in 2007. Each imputation model produced 100 imputed datasets, each with 63,028 rows for the 2007 survey. Compared to the average number of true positives produced by the PHQ-BIN model (*n* = 861), the average number of true positive cases was 87% higher with the PHQ-ORD model (*n* = 1611). Conversely the number of false negative cases were 44% lower (i.e., *n* = 1692 from PHQ-BIN and *n* = 941 from PHQ-ORD model). The ALL-ORD model correctly imputed suicidal ideation, with the highest proportion of accurate matches (66%). Of the imputed models, the PHQ-BIN model had the fewest true positive cases and the most false negative cases (Table 1). The sensitivity improved when 4-level suicidal ideation was used in the models, such that PHQ-ORD and ALL-ORD performed substantially better than PHQ-BIN and ALL-BIN (sensitivity = 63 and 66% vs. 34 and 38%, respectively). Specificity did not appear to change between the imputed models and was consistently above 96%. The PPV improved when 4-level suicidal ideation was used in the PHQ-ORD and ALL-ORD (40 and 42%, respectively) versus the PHQ-BIN and ALL-BIN models (36 and 40%, respectively). When compared to the sensitivities and PPVs for the imputation models with the depression items only (i.e., PHQ-BIN and PHQ-ORD), the inclusion of additional predictors in ALL-BIN and ALL-ORD further improved performance of these statistics, resulting in the ALL-ORD model having the highest sensitivity and PPV. NPV did not appear to change between the imputed models and was consistently above 97%. As expected, the RAN model that did not utilize predictor information with suicidal ideation assigned at random had the lowest sensitivity, specificity, and predictive values. Although this was not the main interest of this study, we observed that the imputation models that utilized binary suicidal ideation (PHQ-BIN and ALL-BIN) underestimated the frequency of endorsed suicidal ideation (3.77 and 3.87%, respectively). Conversely, the imputation models that used a 4-level suicidal ideation (PHQ-ORD and ALL-ORD) overestimated suicidal ideation (6.32 and 6.39%, respectively).

**Table 1:** Diagnostic statistics of imputed suicidal ideation among Millennium Cohort Study participants at 2007 survey, *n* = 63,028

	Self-reported	RAN	PHQ-BIN	PHQ-ORD	ALL-BIN	ALL-ORD
True negative	60,475	58,032 (57,873–58,192)	58,965 (58,815–59,114)	58,103 (57,955–58,251)	59,001 (58,844–59,157)	58,137 (57,985–58,288)
True positive	2553	104 (73–134)	861 (778–944)	1611 (1530–1693)	967 (885–1049)	1689 (1606–1772)
False negative	–	2450 (2352–2548)	1692 (1592–1792)	942 (877–1006)	1587 (1492–1682)	865 (803–927)
False positive	–	2442 (2313–2571)	1510 (1394–1626)	2372 (2257–2487)	1473 (1348–1598)	2338 (2218–2457)
Prevalence	4.05	4.04 (3.79–4.29)	3.77 (3.44–4.08)	6.32 (6.01–6.64)	3.87 (3.55–4.20)	6.39 (6.07–6.71)
Sensitivity	–	4 (3–5)	34 (31–37)	63 (61–65)	38 (35–41)	66 (64–68)
Specificity	–	96 (96–96)	98 (97–98)	96 (96–96)	98 (97–98)	96 (96–96)
PPV	–	4 (3–5)	36 (34–39)	40 (39–42)	40 (37–42)	42 (40–44)
NPV	–	96 (96–96)	97 (97–97)	98 (98–99)	97 (97–98)	99 (98–99)
CPU time	–	–	2:32:48.60	2:26:04.96	70:55:18.03	71:09:58.09

CPU: central processing unit; reported in hours, minutes, seconds; NPV: negative predictive value; PCL-C, PTSD Checklist−Civilian Version; PHQ, Patient Health Questionnaire; PPV: positive predictive value; PTSD, posttraumatic stress disorder

Self-reported suicidal ideation was indicated if reported “several days” or more to “thoughts that you would be better off dead or hurting yourself in some way”

RAN: suicidal ideation was randomly assigned in line with the prevalence observed in the sample in 2007

PHQ-BIN model: treated suicidal ideation as a dichotomous variable and included the remaining 8 PHQ items in the imputation model

PHQ-ORD model: treated suicidal ideation as a 4-level variable and included the remaining 8 PHQ items in the imputation model

ALL-BIN model: treated suicidal ideation as a dichotomous variable and included the 8 PHQ items and previously identified factors from the literature: sex, age, race/ethnicity, marital status, education attainment, 10 individual items from the RAND physical functioning module, 17 individual items and PTSD screener from the PCL-C, smoking status, sleep duration, and 5 alcohol use items from the PHQ [27–30]

ALL-ORD model: treated suicidal ideation as a 4-level variable and included the 8 PHQ items and previously identified factors from the literature: sex, age, race/ethnicity, marital status, education attainment, 10 individual items from the RAND physical functioning module, 17 individual items and PTSD screener from the PCL-C, smoking status, sleep duration, and 5 alcohol use items from the PHQ [27–30]

Among the 63,028 participants, 59.3% participants were never smokers, 26.8% were former smokers and 13.9% were current smokers at the 2007 survey. In the model adjusting for sex, age, race/ethnicity, marital status, and education, suicidal ideation (i.e., actual self-report) was more likely to be endorsed by both former smokers (adjusted odds ratio [AOR] = 1.23, 95% confidence interval [CI]: 1.12–1.36) and current smokers (AOR = 1.87, 95% CI: 1.68–2.08; Table 2) compared with never smokers. Although effect estimates for smoking status with imputed suicidal ideation were in the expected direction for all models (Table 2), the effect estimates were generally higher for imputed suicidal ideation compared with actual self-report. The ALL-BIN model had effect estimates that were closest to the observed self-report associations (AOR for former smoking = 1.23, 95% CI: 1.05–1.44; AOR for current smoking = 1.86, 95% CI: 1.56–2.22). The 95% CIs for the associations between all imputed suicidal ideation with smoking status overlapped with the true AOR 95% CI.

**Table 2:** Associations between suicidal ideation with smoking status at the 2007 survey, the Millennium Cohort Study, *n* = 63,028

	Smoking status (ref: never)^*^	
	Former	Current
	AOR^†^ (95% CI)	AOR^†^ (95% CI)
Self-reported	1.23 (1.12, 1.36)	1.87 (1.68, 2.08)
PHQ-BIN	1.31 (1.15, 1.48)	1.48 (1.62, 2.15)
PHQ-ORD	1.34 (1.23, 1.46)	2.01 (1.83, 2.20)
ALL-BIN	1.23 (1.05, 1.44)	1.86 (1.56, 2.22)
ALL-ORD	1.36 (1.24, 1.48)	2.09 (1.90, 2.29)

All confidence intervals of AORs for the imputed suicidal ideation with smoking status overlapped with the 95% CI for the AOR observed for the self-reported suicidal ideation with smoke status

^*^Never smoker: had not smoked at least 100 cigarettes; former smoker: had smoked at least 100 cigarettes and had successfully quit smoking; current smoker: had smoked at least 100 cigarettes and had not successfully quit

^†^Adjusted for sex, age, race/ethnicity, marital status, and education

AOR, adjusted odds ratio; CI, confidence interval; PCL-C, PTSD Checklist−Civilian Version; PHQ, Patient Health Questionnaire; PTSD, posttraumatic stress disorder

Self-reported suicidal ideation was indicated if reported “several days” or more to “thoughts that you would be better off dead or hurting yourself in some way”

PHQ-BIN model: treated suicidal ideation as a dichotomous variable and included the remaining 8 PHQ items in the imputation model

PHQ-ORD model: treated suicidal ideation as a 4-level variable and included the remaining 8 PHQ items in the imputation model

ALL-BIN model: treated suicidal ideation as a dichotomous variable and included the 8 PHQ items and previously identified factors from the literature: sex, age, race/ethnicity, marital status, education attainment, 10 individual items from the RAND physical functioning module, 17 individual items and PTSD screener from the PCL-C, smoking status, sleep duration, and 5 alcohol use items from the PHQ [27–30]

ALL-ORD model: treated suicidal ideation as a 4-level variable and included the 8 PHQ items and previously identified factors from the literature: sex, age, race/ethnicity, marital status, education attainment, 10 individual items from the RAND physical functioning module, 17 individual items and PTSD screener from the PCL-C, smoking status, sleep duration, and 5 alcohol use items from the PHQ [27–30]

Hours of sleep among the 63,028 participants had the following distribution: 18.2% reported 5 or less hours of sleep, 33.0% reported 6 h of sleep, 46.5% reported 7–9 h of sleep and 2.4% reported 10 or more hours of sleep at the time of the 2007 survey. Adjusting for sex, age, race/ethnicity, marital status, and education, suicidal ideation (i.e., actual self-report) was more likely to be endorsed by those with suboptimal average sleep duration compared to those with an average of 7–9 h of sleep (AOR for 5 h or less = 5.44, 95% CI: 4.87–6.07; AOR for 6 h = 2.08, 95% CI: 1.85–2.33; AOR for 10 or more hours = 6.19, 95% CI: 5.13–7.46; Table 3). The PHQ-BIN model had effect estimates that were closest to the observed self-report associations (AOR for 5 h or less = 5.45, 95% CI: 4.65–6.38; AOR for 6 h = 2.04, 95% CI: 1.74–2.39; AOR for 10 or more hours = 5.35, 95% CI: 4.12–6.93). The 95% CIs for the associations between all imputed suicidal ideation with sleep duration overlapped with the true AOR 95% CI except for the 95% CI produced by the PHQ-ORD and ALL-ORD with sleeping 5 h or less (Table 3).

**Table 3:** Associations between suicidal ideation with sleep duration at the 2007 survey, the Millennium Cohort Study, *n* = 63,028

	Average hours of sleep (ref: 7–9)^a^		
	≤5 AOR^b^ (95% CI)	6 AOR^b^ (95% CI)	≥10 AOR^b^ (95% CI)
Self-reported	5.44 (4.87, 6.07)	2.08 (1.85, 2.33)	6.19 (5.13, 7.46)
PHQ-BIN	5.45 (4.65, 6.38)	2.04 (1.74, 2.39)	5.35 (4.12, 6.93)
PHQ-ORD	6.97 (6.30, 7.71)^c^	2.20 (1.98, 2.44)	7.45 (6.32, 8.79)
ALL-BIN	5.29 (4.51, 6.21)	1.99 (1.70, 2.33)	6.04 (4.75, 7.69)
ALL-ORD	7.16 (6.47, 7.93)^c^	2.20 (1.97, 2.45)	7.83 (6.65, 9.23)

^a^Sleep groups based on National Sleep Foundation recommendations [26]

^b^Adjusted for sex, age, race/ethnicity, marital status, and education

^c^Confidence interval did not overlap with the 95% CI for the AOR observed for the self-reported suicidal ideation with sleep duration

AOR, adjusted odds ratio; CI, confidence interval; PCL-C, PTSD Checklist−Civilian Version; PHQ, Patient Health Questionnaire; PTSD, posttraumatic stress disorder

Self-reported suicidal ideation was indicated if reported “several days” or more to “thoughts that you would be better off dead or hurting yourself in some way”

PHQ-BIN model: treated suicidal ideation as a dichotomous variable and included the remaining 8 PHQ items in the imputation model

PHQ-ORD model: treated suicidal ideation as a 4-level variable and included the remaining 8 PHQ items in the imputation model

ALL-BIN model: treated suicidal ideation as a dichotomous variable and included the 8 PHQ items and previously identified factors from the literature: sex, age, race/ethnicity, marital status, education attainment, 10 individual items from the RAND physical functioning module, 17 individual items and PTSD screener from the PCL-C, smoking status, sleep duration, and 5 alcohol use items from the PHQ [27–30]

ALL-ORD model: treated suicidal ideation as a 4-level variable and included the 8 PHQ items and previously identified factors from the literature: sex, age, race/ethnicity, marital status, education attainment, 10 individual items from the RAND physical functioning module, 17 individual items and PTSD screener from the PCL-C, smoking status, sleep duration, and 5 alcohol use items from the PHQ [27–30]

Among the random 10,000 participants drawn for the MLMI sub-analysis, 3.97% of participants endorsed suicidal ideation as measured by the ninth item on the PHQ-9 in 2007. Comparable counts of average true positives were produced from both of the models (SLMI: 156; MLMI: 153), although these represent less than 40% of those who endorsed suicidal ideation. Additionally, the SLMI and MLMI imputation models resulted in almost identical sensitivities (39% for both models), specificities (98% for both models), PPVs (41 and 42%, respectively), and NPVs (97% for both models) for imputed compared to actual self-reported suicidal ideation (Additional file 1). All AORs between imputed suicidal ideation with smoking status and sleep duration overlapped with self-reported associations for both the SLMI and MLMI (Additional files 2 and 3).

## Discussion

The current study evaluated the efficacy of MI to recover information on suicidal ideation that was completely and intentionally missing due to the removal of the question from a follow-up survey. Suicidal ideation was successfully imputed using limited available additional information. Associations with related constructs (i.e., smoking status and sleep duration) remained for all imputed suicidal ideation models. Post-hoc analyses found that MLMI and SLMI produced comparable imputed values for suicidal ideation in our study design.

Much of the predictive ability observed from the imputation models run in this study was informed by the other items on the PHQ-9. However, depression is strongly related to suicidal ideation, which is part of the diagnostic criteria for depression. Future research should examine whether this finding remains true when predictors have weaker associations with items being imputed. Both of the imputation models that incorporated the 4-level suicidal ideation had the highest numbers of false positives. High proportions of false positives and false negatives led to reduced sensitivities and PPVs for all of the models. The proportion of false negatives was low for all imputation models (below 3%), which, combined with the low prevalence of suicidal ideation, resulted in a greater proportion of true negatives than false and high NPVs. As this misclassification is clearly not ideal when assessing rates among a population, using imputed values of missing variables should not be used to estimate prevalence of rare conditions [33, 34]. Although estimating prevalence of suicidal ideation was not the goal of this analysis, it is notable that the ALL-BIN model produced an average prevalence estimate of 3.87%, which was the closest to the actual prevalence of 4.05% observed on the 2007 survey. The PHQ-ORD and ALL-ORD models, with the most false positives, overestimated prevalence by more than 50%. Different trends in the data may alter the effectivity of the described procedure. For example, simulation data (available upon request) indicated that bias can result from increasing prevalence over time, but that these were minor relative to the overall effect (e.g., bias of 0.08 when Cohen’s d is 2.0). Future research using simulations should be conducted to determine how other factors impact the described procedure.

Consistent with findings from prior studies, we observed that suicidal ideation was positively associated with being a current or former smoker [35] and low and high sleep duration [36, 37]. The associations between self-reported suicidal ideation with smoking status and sleep duration were most closely replicated by the ALL-BIN and PHQ-BIN imputation models, respectively, and were in the expected direction. Using the 4-level suicidal ideation variable inflated the magnitude of the associations for both smoking status and sleep duration which indicates that variables should be imputed in the functional form for which they will be used in analyses.

MLMI should theoretically be advantageous in imputing factors such as suicidal ideation since MLMI takes into account within-subject variations. In our post-hoc analyses comparing MLMI and SLMI, we did not observe a significant improvement in diagnostic statistics or accuracy of adjusted associations between imputed suicidal ideation with related constructs compared to associations observed the self-reported suicidal ideation. One consideration when using the MLMI was that computational limitations made it an unrealistic option for the entire eligible sample of more than 63,000. In unique situations with smaller study populations (e.g., 10,000 or less) where most factors are continuous values, MLMI may be feasible. However, based on our results, MLMI may not be substantially better than SLMI.

Central processing unit time was tracked for the MI models. The time needed to impute these models was not so great that it would be particularly problematic for researchers, with the shortest imputation completed in under 3 h and the longest imputation completing under 3 days (i.e., approximately a weekend of run time). Smaller data sets would be expected to converge to a solution faster than larger data sets. Notably, the ALL-BIN and ALL-ORD models took over 15 times longer to run compared to the PHQ-BIN and PHQ-ORD models, without substantial benefits to diagnostic statistics or improved associations to related constructs, suggesting the payoff may not be worth the time.

This study has notable limitations and strengths. Due to computational limitations, MLMI had to be run among a random subsample of the eligible population with a modified imputation model that contained a reduced number of factors and all factors in a continuous form except for suicidal ideation. Although imputing categorical factors continuously should not adversely affect the imputation, there are potential consequences in subsequent analyses conducted. Our study design used two time points in analyses and the benefits of MLMI might be more apparent with three or more time points. Finally, this study was only able to test the MI method in one study population, which limits the generalizability of the findings.

Notable strengths included the large sample with multiple follow-up surveys of longitudinal survey data that facilitated the assessment of a rare outcome such as suicidal ideation. The study population consisted of participants who were drawn from random samples of roster files in 2000 and 2003 that represent all branches and components of the military and has shown to be representative of the US military [18]. Additionally, a large proportion of survey questions remained consistent across surveys, allowing for the examination of multiple full imputation models.

Our findings demonstrate the utility of imputation of suicidal ideation for use as a covariate in adjusted analyses. The inclusion of this imputed covariate was observed to maintain the correct adjusted associations between outcomes and other predictors in the model (Additional files 4 and 5). As mentioned previously, imputed variables should be in the functional form for which they will be used in analyses. In the context of a longitudinal cohort study such as the source of data for this analysis, the application of this MI method should not be used to estimate prevalence or screen individuals for suicidal ideation. All of the imputation models examined in this study imputed more false positives than true positives. Because suicidal ideation is rare, it is critical to minimize false positives and maximize specificity. To test that the difficulty in imputing suicidal ideation was because of the low prevalence, we applied a similar method as the ALL-BIN imputation model to a more commonly endorsed item on the PHQ-9 depression measure that asks about “feeling tired or having little energy” (endorsed by 42.66% on the 2007 survey). We ran a SLMI model similar to the ALL-BIN but with the tired/little energy item completely missing on the 2007 survey. The sensitivity (95%: 71–72%) and PPV (95%: 70–72%) for imputed feeling tired/little energy out preformed those observed for the imputed suicidal ideation (sensitivity: 95%: 35–41%; PPV: 95%: 37–42%). Conversely, the specificity (95%: 78–79%) and NPV (95%: 78–79%) for imputed feeling tired were not as high as those observed for the imputed suicidal ideation (specificity: 95%: 97–98%; NPV: 95%: 97–98%). This has been observed in previous studies that examined how diagnostic statistics are impacted by prevalence of the outcome [38]. Given the observed bias for more false positives than true positives for suicidal ideation, we would be hesitant to recommend using multiple imputation for suicidal ideation as the outcome of interest. Results demonstrated large amounts of misclassification for individual observations, and thus is likely not a good method for identifying individual characteristics (e.g., for inclusion criteria, follow-up). Other than suicidal ideation, this method could be expanded to other constructs that are not available on all follow-up surveys for a longitudinal study.

## Conclusion

MI is a feasible means for estimating values for factors that were not assessed at a given time point in a longitudinal survey. This technique can benefit longitudinal studies by allowing for such missing constructs to be controlled for in analyses. Because longitudinal studies have to be both adaptable to changing concerns and sensitive to participant burden from survey length, MI is one method to mitigate the impacts of missing data. This method is effective at imputing suicidal ideation in the absence of directly ascertaining it from study participants. Future investigations should be conducted on different missing variables in other longitudinal studies to corroborate these observed results.

## Supplementary information

**Additional file 1 Supplemental Table 1** Diagnostic statistics of imputed suicidal ideation among Millennium Cohort Study participants at 2007 survey, *n* = 10,000.

**Additional file 2 Supplemental Table 2** Associations between suicidal ideation with smoking status at the 2007 survey, the Millennium Cohort Study, *n* = 10,000.

**Additional file 3 Supplemental Table 3** Associations between suicidal ideation with sleep duration at the 2007 survey, the Millennium Cohort Study, *n* = 10,000.

**Additional file 4 Supplemental Table 4** Adjusted associations and 95% confidence intervals between demographic predictors and average hours of sleep, the Millennium Cohort Study, *n* = 63,028.

**Additional file 5 Supplemental Table 5** Associations and 95% confidence intervals between demographic predictors and smoking status, the Millennium Cohort Study, *n* = 63,028.

## Data Availability

The data sets analyzed during the current study are not publicly available due to institutional regulations protecting service member survey responses but are available from the corresponding author upon reasonable request (may require data use agreements).

## Abbreviations

DMDC: Defense Manpower Data Center
DSM-IV: Diagnostic and Statistical Manual of Mental Disorders, Fourth Edition
FCS: Fully conditional specification
IRB: Institutional review board
MI: Multiple imputation
MLMI: Multilevel multiple imputation.
NHANES: National Health and Nutrition Examination Survey
NPV: Negative predictive value
PCL-C: Posttraumatic Stress Disorder Checklist−Civilian Version
PHQ: Patient Health Questionnaire
PPV: Positive predictive value
PTSD: Posttraumatic stress disorder
SLMI: Single-level multiple imputation
VA: Department of Veterans Affairs
