# Supplementary material for: Multiple imputation validation study: addressing unmeasured survey data in a longitudinal design
Source: BMC Med Res Methodol. 2021 Jan 6;21:5. doi: 10.1186/s12874-020-01158-w (PMC7789687; doi:10.1186/s12874-020-01158-w)
Supplement: Supplementary file 1 — Additional file 1 Supplemental Table 1 Diagnostic statistics of imputed suicidal ideation among Millennium Cohort Study participants at 2007 survey, n = 10,000. [file 12874_2020_1158_MOESM1_ESM.docx]

**Supplemental Table 1** Diagnostic statistics of imputed suicidal ideation among Millennium Cohort Study participants at 2007 survey, *n* = 10,000

|  | Self-reported | SLMI | MLMI |
| --- | --- | --- | --- |
| True negative | 9603 | 9378 (9317 – 9438) | 9388 (9331 – 9445) |
| True positive | 397 | 156 (126 – 186) | 153 (122 – 184) |
| False negative | – | 241 (206 – 276) | 244 (208 – 280) |
| False positive | – | 225 (178 – 273) | 215 (172 – 259) |
| Prevalence | 3.97 | 3.81 (3.04 – 4.58) | 3.69 (2.94 – 4.43) |
| Sensitivity | – | 39 (33 – 46) | 39 (32 – 46) |
| Specificity | – | 98 (97 – 98) | 98 (97 – 98) |
| PPV | – | 41 (34 – 48) | 42 (35 – 48) |
| NPV | – | 97 (97 – 98) | 97 (97 – 98) |
| CPU time | – | 2:18:23 | 68:41:37 |

SLMI: single-level multiple imputation; MLMI: multilevel multiple imputation; CPU: central processing unit; reported in hours, minutes, seconds; NPV: negative predictive value; PCL-C, PTSD Checklist−Civilian Version; PHQ, Patient Health Questionnaire; PPV: positive predictive value; PTSD, posttraumatic stress disorder.

Self-reported suicidal ideation was indicated if reported “several days” or more to “thoughts that you would be better off dead or hurting yourself in some way”.

Imputation models treated suicidal ideation as a dichotomous variable and included the 8 PHQ items and previously identified factors from the literature: sex, age, race/ethnicity, marital status, education attainment, 10 individual items from the RAND physical functioning module, 17 individual items and PTSD screener from the PCL-C, smoking status, sleep duration, and 5 alcohol use items from the PHQ [27–30].
