# Supplementary material for: Multiple imputation validation study: addressing unmeasured survey data in a longitudinal design
Source: BMC Med Res Methodol. 2021 Jan 6;21:5. doi: 10.1186/s12874-020-01158-w (PMC7789687; doi:10.1186/s12874-020-01158-w)
Supplement: Supplementary file 2 — Additional file 2 Supplemental Table 2 Associations between suicidal ideation with smoking status at the 2007 survey, the Millennium Cohort Study, n = 10,000. [file 12874_2020_1158_MOESM2_ESM.docx]

**Supplemental Table 2** Associations between suicidal ideation with smoking status at the 2007 survey, the Millennium Cohort Study, *n* = 10,000

|  | Smoking status (ref: never)^*^ | |
| --- | --- | --- |
|  | Former | Current |
|  | AOR^†^ (95% CI) | AOR^†^ (95% CI) |
| Self-reported | 1.23 (0.96, 1.58) | 1.87 (1.43, 2.44) |
| SLMI | 1.18 (0.81, 1.74) | 1.88 (1.26, 2.81) |
| MLMI | 1.14 (0.78, 1.64) | 2.07 (1.43, 3.01) |

All confidence intervals of AORs for the imputed suicidal ideation with smoking status overlapped with the 95% CI for the AOR observed for the self-reported suicidal ideation with smoke status.

^*^Never smoker: had not smoked at least 100 cigarettes; former smoker: had smoked at least 100 cigarettes and had successfully quit smoking; current smoker: had smoked at least 100 cigarettes and had not successfully quit.

^†^Adjusted for sex, age, race/ethnicity, marital status, and education.

AOR, adjusted odds ratio; CI, confidence interval; SLMI: single-level multiple imputation; MLMI: multi-level multiple imputation.

Self-reported suicidal ideation was indicated if reported “several days” or more to “thoughts that you would be better off dead or hurting yourself in some way”.
