# Supplementary material for: Multiple imputation validation study: addressing unmeasured survey data in a longitudinal design
Source: BMC Med Res Methodol. 2021 Jan 6;21:5. doi: 10.1186/s12874-020-01158-w (PMC7789687; doi:10.1186/s12874-020-01158-w)
Supplement: Supplementary file 3 — Additional file 3 Supplemental Table 3 Associations between suicidal ideation with sleep duration at the 2007 survey, the Millennium Cohort Study, n = 10,000. [file 12874_2020_1158_MOESM3_ESM.docx]

**Supplemental Table 3** Associations between suicidal ideation with sleep duration at the 2007 survey, the Millennium Cohort Study, *n* = 10,000

|  | Average hours of sleep (ref: 7–9)^*^ | | |
| --- | --- | --- | --- |
|  | ≤5 | 6 | ≥10 |
|  | AOR^†^ (95% CI) | AOR^†^ (95% CI) | AOR^†^ (95% CI) |
| Self-reported | 5.46 (4.13, 7.23) | 2.27 (1.70, 3.03) | 6.71 (4.24, 10.61) |
| SLMI | 4.85 (3.24, 7.24) | 1.76 (1.17, 2.63) | 5.69 (2.98, 10.87) |
| MLMI | 5.21 (3.44, 7.89) | 1.83 (1.24, 2.72) | 6.12 (3.23, 11.60) |

All confidence intervals of AORs for the imputed suicidal ideation with smoking status overlapped with the 95% CI for the AOR observed for the self-reported suicidal ideation with sleep duration.

^*^Sleep groups based on National Sleep Foundation recommendations [26].

^†^Adjusted for sex, age, race/ethnicity, marital status, and education.

AOR, adjusted odds ratio; CI, confidence interval; SLMI: single-level multiple imputation; MLMI: multi-level multiple imputation.

Self-reported suicidal ideation was indicated if reported “several days” or more to “thoughts that you would be better off dead or hurting yourself in some way”.
