# Supplementary material for: Multiple imputation validation study: addressing unmeasured survey data in a longitudinal design
Source: BMC Med Res Methodol. 2021 Jan 6;21:5. doi: 10.1186/s12874-020-01158-w (PMC7789687; doi:10.1186/s12874-020-01158-w)
Supplement: Supplementary file 4 — Additional file 4 Supplemental Table 4 Adjusted associations and 95% confidence intervals between demographic predictors and average hours of sleep, the Millennium Cohort Study, n = 63,028. [file 12874_2020_1158_MOESM4_ESM.docx]

**Supplemental Table 4** Adjusted associations and 95% confidence intervals between demographic predictors and average hours of sleep, the Millennium Cohort Study, *n* = 63,028

| Suicidal ideation predictor model | Outcome: average hours of sleep  (ref: 7–9)^*^ | Sex  (ref: male) | Age | Race/ ethnicity  (ref: white, non-Hispanic) | | Marital status  (ref: single) | | Education |
| --- | --- | --- | --- | --- | --- | --- | --- | --- |
|  |  | Female | 1-unit increase | Black, non-Hispanic | Other | Married | Previously married | 1-unit increase |
| Self-reported AOR^†^ | ≤5 | 0.93 (0.88, 0.98) | 0.98 (0.97, 0.98) | 2.06 (1.93, 2.21) | 1.47 (1.38, 1.56) | 1.18 (1.10, 1.26) | 1.82 (1.68, 1.97) | 0.77 (0.76, 0.79) |
|  | 6 | 0.84 (0.80, 0.87) | 0.99 (0.99, 0.99) | 1.44 (1.36, 1.53) | 1.28 (1.22, 1.35) | 1.15 (1.09, 1.21) | 1.41 (1.31, 1.51) | 0.93 (0.91, 0.94) |
|  | ≥10 | 2.02 (1.81, 2.27) | 0.98 (0.97, 0.99) | 2.34 (2.04, 2.69) | 1.30 (1.12, 1.51) | 0.71 (0.62, 0.81) | 1.13 (0.96, 1.33) | 0.65 (0.62, 0.68) |
| PHQ-BIN AOR^†^ | ≤5 | 0.91 (0.87, 0.96) | 0.98 (0.97, 0.98) | 2.07 (1.93, 2.21) | 1.47 (1.39, 1.56) | 1.15 (1.08, 1.23) | 1.79 (1.65, 1.94) | 0.77 (0.76, 0.79) |
|  | 6 | 0.83 (0.80, 0.87) | 0.99 (0.99, 0.99) | 1.44 (1.36, 1.53) | 1.28 (1.22, 1.35) | 1.14 (1.08, 1.21) | 1.40 (1.31, 1.50) | 0.93 (0.91, 0.94) |
|  | ≥10 | 2.00 (1.78, 2.23) | 0.98 (0.97, 0.99) | 2.34 (2.04, 2.69) | 1.31 (1.13, 1.52) | 0.69 (0.61, 0.79) | 1.12 (0.95, 1.32) | 0.65 (0.62, 0.68) |
| PHQ-ORD AOR^†^ | ≤5 | 0.91 (0.86, 0.96) | 0.98 (0.97, 0.98) | 2.08 (1.94, 2.23) | 1.46 (1.37, 1.55) | 1.17 (1.10, 1.25) | 1.74 (1.60, 1.89) | 0.78 (0.77, 0.80) |
|  | 6 | 0.83 (0.80, 0.87) | 0.99 (0.99, 0.99) | 1.44 (1.36, 1.53) | 1.28 (1.22, 1.34) | 1.15 (1.09, 1.21) | 1.39 (1.30, 1.49) | 0.93 (0.92, 0.94) |
|  | ≥10 | 1.98 (1.77, 2.22) | 0.98 (0.98, 0.99) | 2.37 (2.06, 2.72) | 1.30 (1.12, 1.51) | 0.70 (0.62, 0.81) | 1.08 (0.92, 1.28) | 0.66 (0.62, 0.69) |
| ALL-BIN AOR^†^ | ≤5 | 0.92 (0.87, 0.97) | 0.97 (0.97, 0.98) | 2.03 (1.89, 2.17) | 1.45 (1.37, 1.54) | 1.18 (1.10, 1.26) | 1.82 (1.67, 1.97) | 0.78 (0.76, 0.79) |
|  | 6 | 0.83 (0.80, 0.87) | 0.99 (0.99, 0.99) | 1.40 (1.31, 1.48) | 1.26 (1.20, 1.32) | 1.15 (1.09, 1.21) | 1.40 (1.31, 1.50) | 0.93 (0.92, 0.95) |
|  | ≥10 | 2.00 (1.78, 2.23) | 0.98 (0.97, 0.99) | 2.26 (1.97, 2.60) | 1.28 (1.10, 1.48) | 0.72 (0.63, 0.82) | 1.14 (0.96, 1.34) | 0.65 (0.62, 0.69) |
| ALL-ORD AOR^†^ | ≤5 | 0.91 (0.87, 0.96) | 0.98 (0.97, 0.98) | 2.05 (1.91, 2.20) | 1.45 (1.36, 1.54) | 1.19 (1.12, 1.27) | 1.75 (1.61, 1.90) | 0.79 (0.77, 0.80) |
|  | 6 | 0.83 (0.80, 0.87) | 0.99 (0.99, 0.99) | 1.40 (1.32, 1.49) | 1.26 (1.20, 1.32) | 1.15 (1.09, 1.21) | 1.39 (1.30, 1.49) | 0.93 (0.92, 0.95) |
|  | ≥10 | 1.98 (1.77, 2.22) | 0.98 (0.97, 0.99) | 2.29 (1.99, 2.63) | 1.28 (1.10, 1.48) | 0.72 (0.63, 0.82) | 1.09 (0.93, 1.29) | 0.66 (0.63, 0.69) |

*Sleep groups based on National Sleep Foundation recommendations [26].

^†^Adjusted for suicidal ideation, sex, age, race/ethnicity, marital status, and education.

AOR, adjusted odds ratio; OR, odds ratio. PCL-C, PTSD Checklist−Civilian Version; PHQ, Patient Health Questionnaire; PTSD, posttraumatic stress disorder.

Self-reported suicidal ideation was indicated if reported “several days” or more to “thoughts that you would be better off dead or hurting yourself in some way”.

PHQ-BIN model: treated suicidal ideation as a dichotomous variable and included the remaining 8 PHQ items in the imputation model.

PHQ-ORD model: treated suicidal ideation as a 4-level variable and included the remaining 8 PHQ items in the imputation model.

ALL-BIN model: treated suicidal ideation as a dichotomous variable and included the 8 PHQ items and previously identified factors from the literature: sex, age, race/ethnicity, marital status, education attainment, 10 individual items from the RAND physical functioning module, 17 individual items and PTSD screener from the PCL-C, smoking status, sleep duration, and 5 alcohol use items from the PHQ [27–30].

ALL-ORD model: treated suicidal ideation as a 4-level variable and included the 8 PHQ items and previously identified factors from the literature: sex, age, race/ethnicity, marital status, education attainment, 10 individual items from the RAND physical functioning module, 17 individual items and PTSD screener from the PCL-C, smoking status, sleep duration, and 5 alcohol use items from the PHQ [27–30].
