# Supplementary material for: Multiple imputation validation study: addressing unmeasured survey data in a longitudinal design
Source: BMC Med Res Methodol. 2021 Jan 6;21:5. doi: 10.1186/s12874-020-01158-w (PMC7789687; doi:10.1186/s12874-020-01158-w)
Supplement: Supplementary file 5 — Additional file 5 Supplemental Table 5 Associations and 95% confidence intervals between demographic predictors and smoking status, the Millennium Cohort Study, n = 63,028. [file 12874_2020_1158_MOESM5_ESM.docx]

**Supplemental Table 5** Associations and 95% confidence intervals between demographic predictors and smoking status, the Millennium Cohort Study, *n* = 63,028

| Suicidal ideation predictor model | Outcome:  smoking status  (ref: never smoker) | Sex  (ref: male) | Age | Race/ethnicity  (ref: white, non-Hispanic) | | Marital status  (ref: single) | | Education |
| --- | --- | --- | --- | --- | --- | --- | --- | --- |
|  |  | Female | 1-unit increase | Black, non-Hispanic | Other | Married | Previously married | 1-unit increase |
| Self-reported AOR* | Former | 1.00 (0.96, 1.05) | 1.02 (1.02, 1.03) | 0.35 (0.33, 0.38) | 0.72 (0.68, 0.76) | 1.20 (1.13, 1.27) | 1.35 (1.25, 1.45) | 0.70 (0.71, 0.73) |
|  | Current | 0.87 (0.82, 0.92 | 1.00 (1.00, 1.00) | 0.44 (0.40, 0.47) | 0.58 (0.53, 0.62) | 0.86 (0.81, 0.93) | 1.50 (1.38, 1.63) | 0.52 (0.51, 0.54) |
| PHQ-BIN AOR* | Former | 1.00 (0.96, 1.05) | 1.02 (1.02, 1.03) | 0.35 (0.33, 0.38) | 0.72 (0.68, 0.75) | 1.20 (1.13, 1.27) | 1.34 (1.25, 1.45) | 0.72 (0.71, 0.73) |
|  | Current | 0.87 (0.82, 0.92) | 1.00 (1.00, 1.00) | 0.44 (0.40, 0.47) | 0.58 (0.53, 0.62) | 0.86 (0.80, 0.92) | 1.49 (1.37, 1.62) | 0.52 (0.51, 0.54) |
| PHQ-ORD AOR* | Former | 1.00 (0.96, 1.04) | 1.02 (1.02, 1.03) | 0.35 (0.33, 0.38) | 0.71 (0.68, 0.75) | 1.20 (1.13, 1.27) | 1.34 (1.24, 1.44) | 0.72 (0.71, 0.73) |
|  | Current | 0.86 (0.82, 0.92) | 1.00 (1.00, 1.00) | 0.43 (0.40, 0.47) | 0.57 (0.53, 0.62) | 0.86 (0.80, 0.92) | 1.47 (1.35, 1.60) | 0.53 (0.51, 0.54) |
| ALL-BIN AOR* | Former | 1.00 (0.96, 1.05) | 1.02 (1.02, 1.03) | 0.35 (0.33, 0.38) | 0.72 (0.68, 0.76) | 1.20 (1.13, 1.27) | 1.35 (1.26, 1.45) | 0.72 (0.70, 0.73) |
|  | Current | 0.87 (0.82, 0.92) | 1.00 (1.00, 1.00) | 0.43 (0.40, 0.47) | 0.57 (0.53, 0.62) | 0.86 (0.81, 0.92) | 1.50 (1.38, 1.63) | 0.52 (0.51, 0.54) |
| ALL-ORD AOR* | Former | 1.00 (0.96, 1.05) | 1.02 (1.02, 1.03) | 0.35 (0.33, 0.38) | 0.71 (0.68, 0.75) | 1.20 (1.14, 1.28) | 1.34 (1.25, 1.44) | 0.72 (0.71, 0.73) |
|  | Current | 0.86 (0.82, 0.92) | 1.00 (1.00, 1.00) | 0.43 (0.40, 0.47) | 0.57 (0.53, 0.62) | 0.87 (0.81, 0.93) | 1.47 (1.36, 1.60) | 0.53 (0.52, 0.54) |

^*^Never smoker: had not smoked at least 100 cigarettes; former smoker: had smoked at least 100 cigarettes and had successfully quit smoking; current smoker: had smoked at least 100 cigarettes and had not successfully quit.

^†^Adjusted for sex, age, race/ethnicity, marital status, and education.

AOR, adjusted odds ratio; CI, confidence interval; PCL-C, PTSD Checklist−Civilian Version; PHQ, Patient Health Questionnaire; PTSD, posttraumatic stress disorder.

Self-reported suicidal ideation was indicated if reported “several days” or more to “thoughts that you would be better off dead or hurting yourself in some way”.

PHQ-BIN model: treated suicidal ideation as a dichotomous variable and included the remaining 8 PHQ items in the imputation model.

PHQ-ORD model: treated suicidal ideation as a 4-level variable and included the remaining 8 PHQ items in the imputation model.

ALL-BIN model: treated suicidal ideation as a dichotomous variable and included the 8 PHQ items and previously identified factors from the literature: sex, age, race/ethnicity, marital status, education attainment, 10 individual items from the RAND physical functioning module, 17 individual items and PTSD screener from the PCL-C, smoking status, sleep duration, and 5 alcohol use items from the PHQ [27–30].

ALL-ORD model: treated suicidal ideation as a 4-level variable and included the 8 PHQ items and previously identified factors from the literature: sex, age, race/ethnicity, marital status, education attainment, 10 individual items from the RAND physical functioning module, 17 individual items and PTSD screener from the PCL-C, smoking status, sleep duration, and 5 alcohol use items from the PHQ [27–30].
